# Trachomatous trichiasis surgeons appreciate using HEAD START for extended training during periods of low surgical activity: A preliminary study

**DOI:** 10.1371/journal.pntd.0013948

**Published:** 2026-02-26

**Authors:** Samantha E. Tulenko, Belay Bayissasse, Fisseha Admassu, Wondu Alemayehu, Alemayehu Sisay, Demissie Tadesse, Emily W. Gower

**Affiliations:** 1 Department of Epidemiology, Gillings School of Global Public Health, University of North Carolina at Chapel Hill, Chapel Hill, North Carolina, United States of America; 2 Orbis International Ethiopia, Addis Ababa, Ethiopia; 3 Department of Ophthalmology, University of Gondar, Gondar, Ethiopia; 4 Berhan Public Health and Eye Care Consultancy, Addis Ababa, Ethiopia; 5 CBM International, Addis Ababa, Ethiopia; Yale University School of Medicine, UNITED STATES OF AMERICA

## Abstract

**Background:**

Trachomatous trichiasis (TT) surgeons routinely experience periods of low surgical activity, which may contribute to low or variable surgical skill. Surgeons need strategies for maintaining skill during these periods.

**Methodology/Principal findings:**

We recruited 28 newly-trained TT surgeons for this pilot study in southern Ethiopia; we randomized 15 TT surgeons to receive extended surgical practice (Extended HEAD START or EHS group) and feedback using the HEAD START simulation device and 13 TT surgeons to follow standard practice (Standard HEAD START or SHS group) during a 5-month period of low surgical activity. A masked external examiner assessed surgical skill during two live surgeries before and after study activities. During the intervention period, three ophthalmologist trainers evaluated EHS surgeon skill on the simulation device and provided feedback monthly. Surgeons and trainers completed questionnaires on the acceptability and utility of extended HEAD START training. Additionally, we compared change in surgeon skill between baseline and follow-up live surgical assessments between EHS and SHS surgeons. On the final questionnaire, 93% of surgeons reported that extended HEAD START training was beneficial and should be implemented as continued education for trained surgeons. In this small pilot study, on average EHS surgeon skill improved across the 5-month period.

**Conclusions/Significance:**

Extended practice with HEAD START is a promising strategy for refining and maintaining surgeon skill during periods of low surgical activity. More research is needed to elicit the most beneficial components of an extended training program and to address logistical challenges.

## Author summary

Globally, an estimated 1.5 million people are living with the blinding condition known as trachomatous trichiasis (TT). This condition occurs when repeated ocular infection causes the eyelid to scar and turn inwards and the eyelashes to touch the eye. In most settings, nonphysician healthcare workers are trained to perform the eyelid surgery that corrects TT. After initial training, these “TT surgeons” operate independently and often have long periods of low surgical volume. This preliminary study investigates TT surgeons’ response to using a surgical simulation device known as HEAD START to continue practicing surgical skills after their initial training. We also assessed whether additional practice and feedback using the HEAD START device helps TT surgeons maintain and improve surgical skills. After five months, almost all surgeons felt it was beneficial to incorporate regular surgical simulator practice as continued education.

## Introduction

In countries where trachoma remains endemic, surgery to correct trachomatous trichiasis (TT) is a key tenet of the SAFE strategy for trachoma elimination. At present, an estimated 1.5 million people around the world are living with TT [1]. To address this large disease burden, countries are scaling up their TT surgery programs. Because the highest trachoma burden is found in countries without extensive access to ophthalmologists or surgeons, trichiasis surgeries are often performed by trained nonphysician health workers [2]. These eye care workers, henceforth referred to as “TT surgeons”, participate in a 2–4-week training that includes classroom instruction, a 3-hour session with the HEAD START surgical simulation device, and supervised surgical practice before they begin performing surgeries in the community [3].

Of concern, patient outcomes from trichiasis surgery remain highly variable [4]. Poor surgical quality is linked to increased rates of post-operative trichiasis [5–7]. One strategy for improving surgeon skill and, in turn, patient outcomes is expanding simulation practice for surgeons [8]. Simulation practice is a routine component of trainings across various surgical fields in high-income countries. Numerous studies have established the potential benefit of simulation training for acquiring surgical skills [9–11]. When combined with evaluation and mentoring, simulators provide a safe environment for surgeons to hone their skills.

To bridge the training gap between the classroom and operating on live TT surgery patients, our team introduced the Human Eyelid Analogue Device for Surgical Training and skill Reinforcement in Trachoma (HEAD START) device to trachoma surgery [8]. This surgical simulation device allows surgery to be practiced in a safe environment while incorporating feedback opportunities and self-directed learning and assessment. In most settings, HEAD START is utilized once during training between classroom and live-surgery practice. Then, surgeons progress to live surgery and typically do not return to the simulator. HEAD START is currently utilized during initial TT surgery training throughout Africa. The device is produced by Ho’s Art LLC (hosartllc@gmail.com), and the company is currently able to meet the global demand for HEAD START supplies.

We are interested in determining whether regular practice with HEAD START provides benefits for newly-trained surgeons, particularly since many surgeons operate seasonally, with long periods of downtime between surgical camps and with limited field supervision. In this preliminary study, we assessed surgeon satisfaction with extended practice on the HEAD START training device during a period of low surgical activity. We compared changes in surgical skill over this period between newly-trained surgeons who had extended practice on HEAD START and surgeons who did not.

## Methods

### Ethics statement

Approval to conduct this study was granted by the Institutional Review Board at the University of North Carolina, Chapel Hill (17–0423) and the Southern Nations Nationalities and People’s State Health Bureau in Ethiopia. Formal consent was obtained in writing in the local language, Amharic by Ethiopian study staff. The study was registered at clinicaltrials.gov (NCT03135080) on April 11, 2017.

### Study population

All new TT surgeons trained by Orbis International in the Wolaita, Gamo Goffa and Gurage districts of the South and Central Ethiopia Regional States (formerly the Southern Nations, Nationalities, and Peoples’ Region) of Ethiopia in April and May 2017 were invited to participate in the study. Orbis’ standard TT surgeon training involves one week of classroom instruction followed by a three-hour, one-on-one HEAD START training session and two weeks of surgical practice under a trainer’s supervision [8]. At the end of this training, half of these newly-trained surgeons were randomly selected to participate in extended HEAD START (EHS) training from May 2017 through October 2017. The remainder served as a control group, referred to as “standard HEAD START” (SHS). An external data analyst used the RAND() function in Excel to randomize trainees to each group.

All TT surgeons were instructed to follow Orbis’ instructions for performing TT surgery through the enhanced training period (May 2017 to October 2017) as usual. Any surgeries performed during this time were recorded at local health centers, per Orbis protocol.

### Intervention

#### Extended HEAD START training.

Participants who were selected for extended HEAD START (EHS) training were randomly assigned to one of three senior ophthalmologist trainers (WA, AS, DT) for the duration of the study. At study initiation, each new surgeon met their trainer and performed one or two live surgeries under the supervision of their trainer. This was an opportunity for trainers to establish rapport with their surgeons and deliver initial feedback on surgical technique. At this meeting, we provided TT surgeons with a HEAD START base and eyelid cartridges to use for practice during the study period. Surgeons identified whether they would perform bilamellar tarsal rotation (BLTR) or posterior lamellar tarsal rotation (PLTR) surgeries for the duration of the project.

For 20 weeks immediately following this meeting, each surgeon performed two TT surgeries on the HEAD START device weekly. Every four weeks, the surgeons mailed eight completed cartridges to their assigned trainer. Trainers completed a standardized assessment of the cartridges, including ranking four skills on a five-point scale and providing recommendations on areas on which to focus for the next month (Form A). Skills evaluated monthly included the ability to: make a straight incision, take proper bites, evenly space sutures, and tie knots. Trainers were asked to conduct monthly phone calls with each surgeon to review the cartridges and provide feedback.

### Evaluation

At baseline (before the intervention) and at the completion of the intervention, an independent examiner (FA) who was not involved in initial surgeon training or the extended HEAD START trainings and was masked to training assignment conducted surgical skills assessments on all study participants. The examiner evaluated each surgeon’s performance on 1–2 live surgeries in May 2017 and at least one live surgery in October 2017. At both assessments, he recorded his assessment on a standardized form (Form B), established previously for evaluation of surgeons when using the HEAD START device in surgical training [8]. This form focused on skill level for each of the critical aspects of TT surgery. It included the four criteria assessed during the monthly cartridge reviews plus criteria that could only be assessed in person, including ability to: place the TT clamp or the Trabut plate properly, hold and manipulate instruments, follow appropriate logical order and technique, and comprehend and implement instructions. Each skill was assessed on a five-point scale. The examiner separately graded surgeons on overall innate skill level.

### Final questionnaire

At the end of the intervention, participants in the extended HEAD START training completed a questionnaire on the utility of regular simulation practice (Form C). Trainers completed a questionnaire on the impact of regular simulation practice on their surgeons’ improvement (Form D). Surgeon questionnaires were completed in Amharic and translated to English; trainer questionnaires were completed in English.

### Analysis

We evaluated changes in individual surgical performance using baseline and final surgical evaluations as well as monthly cartridge evaluations. To evaluate overall changes in surgeon skill over the study period, we compared the sums of scores across all skill levels at baseline and final examinations. To assess the impact of the extended HEAD START intervention, we only considered the four skills that were also evaluated monthly. Thus, each surgeon could receive up to 20 points at each evaluation. Surgeons reported the number of live surgeries performed during the study period, and changes in individual performance were compared across four categories of surgical productivity (<20 surgeries, 21–50 surgeries, 51–100 surgeries, > 100 surgeries). We used descriptive statistics to compare the surgical performance of EHS participants with the surgical performance for SHS surgeons.

We characterized trainee responses to open-ended questions on the final questionnaire regarding the utility of extended HEAD START training and suggestions for future improvements. Similarly, we assessed trainer satisfaction with the extended HEAD START program using responses to the final trainer questionnaire. We coded both questionnaire responses based on common themes and analyzed themes to understand trainee and trainer opinions on the utility of extended HEAD START training.

## Results

### Study participants

**Fig 1:**
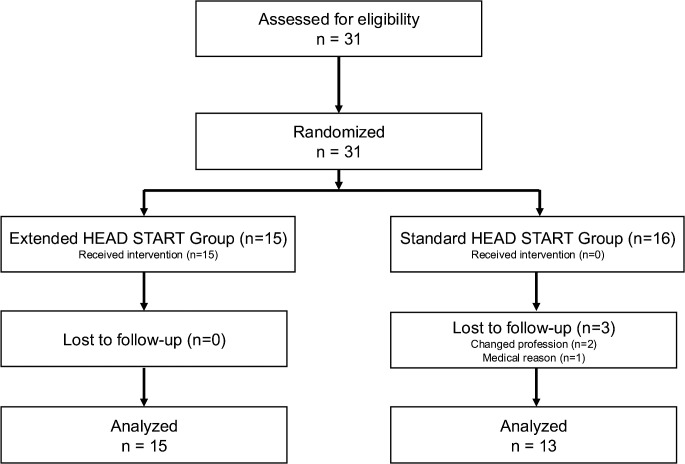
Flow diagram of study enrollment and follow-up.

We enrolled 31 newly-trained surgeons from three trichiasis surgery trainings (Fig 1). After initial evaluations, 15 surgeons were randomized to the extended HEAD START training group, and 16 served as standard HEAD START controls. Three surgeons dropped out. These individuals were male (n = 3) and from Wolaita (2) and Gama Goffa (1). All three are excluded from the final assessment and analysis.

Of the 28 surgeons ultimately included, 15 participated in the extended HEAD START training. Surgeon characteristics were similar between groups (Table 1). Most surgeons were male and operating in Gamo Goffa zone. In both groups, around half of the surgeons conducted 20 or fewer live surgeries over the six-month study period. The majority of surgeons performed the posterior lamellar tarsal rotation procedure (PLTR); only one surgeon elected to perform bilamellar tarsal rotation (BLTR).

**Table 1:** Surgeon Characteristics, by HEAD START Training Assignment.

Characteristics	Extended HEAD START n (%)	Standard HEAD START n (%)
Overall	15 (53.6)	13 (46.4)
Male	13 (86.7)	10 (76.9)
Zone (%)		
Gamo Goffa	6 (40.0)	8 (61.5)
Gurage	4 (26.7)	2 (15.4)
Wolaita	5 (33.3)	3 (23.1)
Live surgeries performed* (%)		
≤ 20 surgeries	7 (46.7)	6 (50.0)
21–50 surgeries	4 (26.7)	2 (16.7)
51–100 surgeries	2 (13.3)	3 (25.0)
> 100 surgeries	2 (13.3)	1 (8.3)
Missing	0	1
Type of surgery (%)		
Bilamellar Tarsal Rotation	0	1 (7.7)
Posterior Lamellar Tarsal Rotation	15 (100)	12 (92.3)

* Numbers reflect live surgeries conducted during the enhanced training period (April 2017 – October 2017).

### Baseline live-surgery evaluations

**Fig 2:**
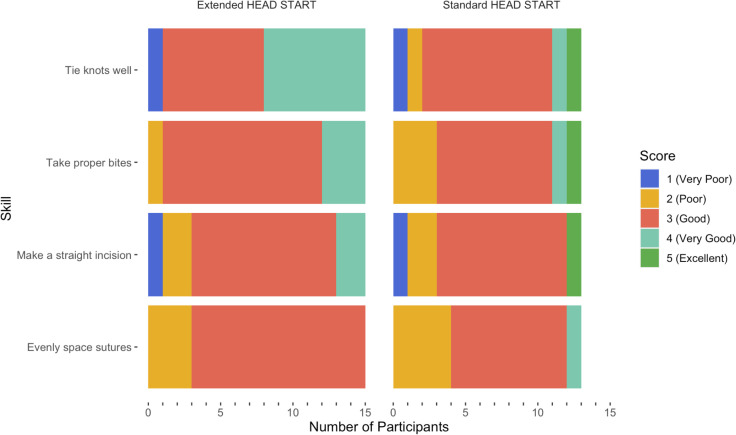
Distribution of surgeon scores at baseline across four surgical skills by surgeon category.

At baseline, overall surgeon aptitude ranged from very low to highly-skilled (Table 2). Most surgeons (64%) were scored as having “about average” overall innate skill. Three surgeons were scored as “very low skill” level, and one surgeon was considered exceptionally skilled. This distribution was similar when surgeons were scored individually on four technical skills (Fig 2). The median score on each of the three skills was a 3 (good). In each group, surgeons received the lowest scores in tying knots well and making a straight incision. A larger proportion of the EHS surgeons scored very good or excellent on knot tying. When all technical skill scores were considered together, the average baseline total score was 11.9 (range: 6–19) and was similar between EHS surgeons (12.1) and control surgeons (11.6).

**Table 2:** Changes in total surgical skill scores and innate skill scores between baseline and final evaluations, by surgeon category.

Summed surgical skill scores* (max score = 20)						
	EHS Surgeons (n = 15)			SHS Surgeons (n = 13)		
	Baseline	Final	Change	Baseline	Final	Change
**Mean**	12.1	13.4	1.3	11.6	12.2	0.5
**Median**	12.0	14.0	2.0	12.0	13.0	1.0
**Range**	6.0-14.0	10.0-16.0	-3.0-8.0	6.0-19.0	7.0-16.0	-9.0-4.0
**Overall innate surgeon skill score** ^†^						
	**EHS Surgeons**			**SHS Surgeons**		
	**Baseline**	**Final**	**Change**	**Baseline**	**Final**	**Change**
**Mean**	3.1	3.5	0.5	2.9	3.1	0.2
**Median**	3.0	3.0	0	3.0	3.0	1.0
**Range**	1.0-5.0	2.0-5.0	-2.0-2.0	1.0-6.0	1.0-5.0	-4.0-2.0

Abbreviations: EHS: Extended HEAD START, SHS: Standard HEAD START.

* Subset evaluated monthly (ability to: make a straight incision, take proper bites, evenly space sutures, and tie knots).

† Overall innate surgeon skill was scored as follows: 1: very low skill level, 2: below average, 3: about average, 4: top 25%, 5: top 10%, 6: the best I have ever seen.

### Monthly surgeon evaluations

One ophthalmologist trainer was unavailable for consistent monthly feedback and conducted only two of the five phone call sessions as part of the EHS intervention. This trainer’s five surgeons continued to practice on the HEAD START device and submit monthly cartridges throughout the extended training. Assessment of monthly scores excludes the 5 EHS surgeons whose trainer was not available for consistent feedback. Sensitivity analyses including these five EHS surgeons were congruent with primary results.

**Fig 3:**
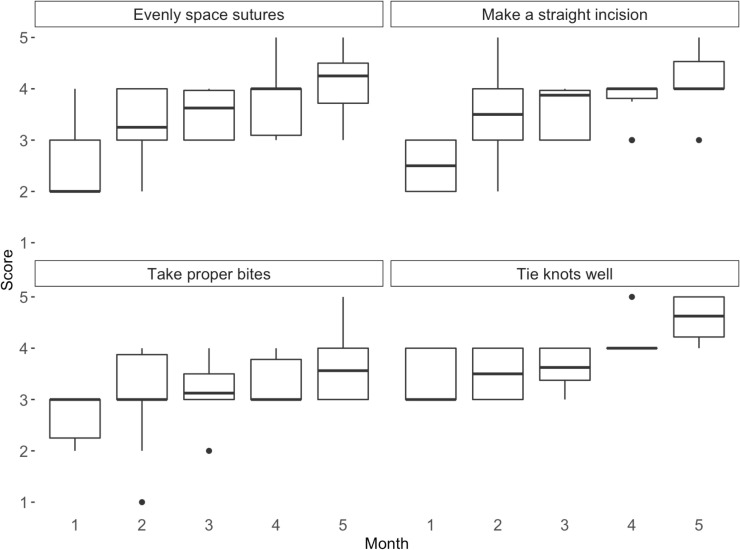
Distribution of surgeon* scores^†^ each month for each surgical skill. * Scores are presented for 10 Extended HEAD START surgeons who received monthly trainer feedback. † Surgeons were scored on a scale ranging from 1 (very poor) to 5 (excellent) for each skill.

At the first month’s evaluation of cartridges, mean scores for each skill area ranged from 2.5-3.4, indicating that surgeons generally had room to enhance their performance on all surgical skills. Half of the surgeons were identified as making “poor” incisions and six were identified as having “poor” suture alignment technique during the first monthly cartridge assessment (Fig 3).

We observed an improvement in scores in each surgical skill across the five months of HEAD START practice, with a mean improvement of 1.4 points (range 0–3) (Fig 3). From month one to month five, every surgeon’s scores improved in almost every focus area. Three exceptions were two surgeons who remained “good” at ability to take proper bites and one surgeon who remained “very good” at suture alignment. Each surgeon’s mean change across all skills ranged from 0.7-2.2. Surgeons saw the greatest improvement (+1.7 points, on average) in incision skills and suture alignment technique. In months four and five, all surgeons scored “good” or better on each skill.

### Final live-surgery evaluations

At the final live-surgery evaluation, most surgeons in both groups received higher overall scores than at baseline evaluations (Table 2), with most receiving “good” to “very good” for each surgical skill. However, the magnitude of surgeon improvements varied substantially between individuals. Total final scores ranged from 10 to 16 (mean 13.4) in the EHS group and 7–16 (mean 12.2) in the SHS group. Change in total scores ranged from -3–8 points in the EHS group and -9–4 in the SHS group.

**Fig 4:**
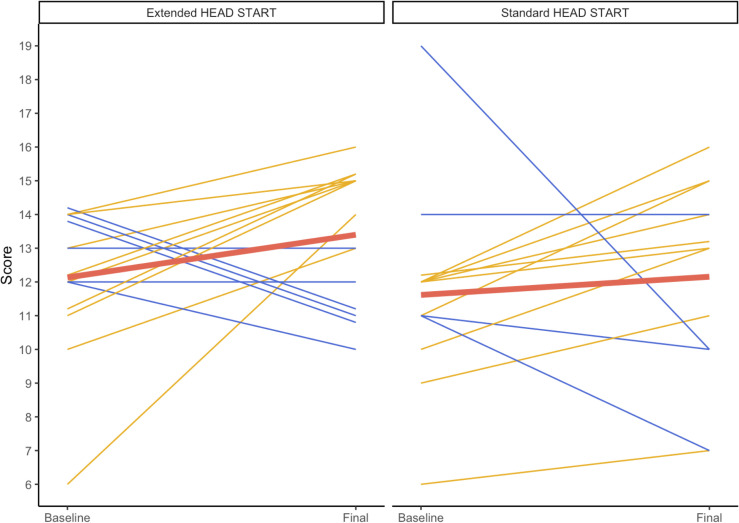
Change in individual surgeon total live surgery skill score from live surgery baseline score to final score for skills* that were specifically evaluated monthly. * Skills evaluated monthly include the ability to: make a straight incision, take proper bites, evenly space sutures, and tie knots. † Overlapping lines where multiple surgeons shared the same baseline and final scores have been offset to aid in visualization. ‡ Red line represents trendline for each plot.

Participants in the EHS intervention had somewhat greater improvement in total scores than SHS surgeons at the final examination (Fig 4), despite EHS surgeons having a slightly higher average baseline live-surgery. The average change from baseline total score to final total score was 1.3 for EHS surgeons and 0.5 for SHS surgeons on the absolute scale (Table 2). Four surgeons in the EHS group (26.7%) and three SHS surgeons (23.1%) had lower total scores at their final evaluation than at their baseline evaluation.

On average, surgeons reported conducting 40 surgeries over the five-month study period (median: 25, range: 0–183) (Table 1). There was no clear pattern of association between the number of live surgeries performed and the surgeon’s final evaluation score or the surgeon’s change in total score from baseline to final evaluation (Table 3). Notably, nearly half of the surgeons in each group performed fewer than 20 surgeries, making assessment of the potential association challenging.

**Table 3:** Final live surgery evaluation scores and changes in total scores by number of live surgeries reported during the study period.

Number of live surgeries performed between baseline and follow-up evaluation	Average final live surgery evaluation score				Average change in total evaluation score between baseline and follow-up evaluation	
	EHS Surgeons		SHS Surgeons			
	N	Score	N	Score	EHS Surgeons	SHS Surgeons
< 20	7	13.3	6	12.5	1.29	1.33
20-50	4	13.2	3	10.5	0.5	0.5
51-100	2	15	2	12.3	1.5	-1.33
>100	2	12.5	1	15	2.5	3
Missing total surgery count			1	10		-1

Abbreviations: EHS: Extended HEAD START, SHS: Standard HEAD START.

### Surgeon satisfaction

When asked, “During periods when you are regularly performing surgery, do you feel it is useful to practice with HEAD START?” the majority of surgeons (93%) indicated that HEAD START practice was beneficial. Summarized surgeon responses to the open-ended final questionnaire are presented in Table 4. Surgeons overwhelmingly felt HEAD START improved their surgical competence, deepened their knowledge of the procedure, and built confidence. When asked for feedback on how to improve the process, surgeons commonly recommended small improvements to the HEAD START device, such as adding eyelashes or using a more pliable material.

**Table 4:** Trainee responses to open-ended final questionnaire.

Questions/responses	Number of trainees (out of 15)
**What did you like about practicing regularly on HEAD START?**	
Improved competence/ knowledge	10
Improved confidence	4
Provided practice that is not live surgery	3
Improved speed	2
It is a good device	1
**What did you dislike about practicing regularly on HEAD START?**	
Device could be improved	9
Too different from live surgery	3
Time demands of the training	3
Nothing	2
**Did you find the monthly conference calls useful? Explain your answer.**	
Yes	15
They helped me improve my skill	8
They provided additional education	4
They improved the experience of using HEAD START	1
**What things should we change about the process?**	
Improve the device*	9
Provide payments for time and transportation	3
Nothing should be changed	3
Provide more practice	1
Decrease the time commitment	1

*Primarily through making the material more pliable and adding eyelashes.

### Trainer satisfaction

The three ophthalmologist trainers echoed the positive feedback from the study surgeons. All three trainers responded that monthly conference calls were useful. Trainers felt that surgeons were enthusiastic and eager to improve while they were getting one-on-one feedback during the study. Additionally, the trainers offered suggestions to improve the extended training program. In particular, trainers thought surgeons could benefit from recording a video of their simulation surgery and sharing it with the trainer. Recording the procedure would allow trainers to provide feedback on aspects of surgery that could not be evaluated from a cartridge alone, such as instrument handling.

## Discussion

This pilot study highlighted that TT surgeons and their trainers believe extended use of the HEAD START surgical simulator [8] is beneficial for improving surgeon skill during a period of typically low workload. Surgeons who utilized the device regularly reported feeling more knowledgeable, confident, and efficient after practicing weekly with HEAD START for five months. Additionally, they felt that feedback from their trainers during conference calls was constructive. Repeatedly, surgeons expressed that this extended practice should become standard in TT surgery trainings. Though results are preliminary and statistical power is limited by small sample size, this feedback highlights the importance of continued engagement with surgeons after the initial training, particularly during periods when they are still refining their skills.

TT surgery trainings produce community eye care workers who can perform sight-preserving surgery. While training capabilities have improved over time, surgeon skill is highly variable upon completion of training [12]. Our baseline live-surgery evaluations suggest that most surgeons would benefit from additional supervised practice. We conducted baseline evaluations at the point when surgeons were beginning to perform live surgeries independently, yet seven surgeons were flagged as having low to very low skill level. Without an option for additional independent practice, these surgeons with low skill may develop long-term surgical habits with potentially harmful consequences for patients. There is a need for extended training options for the weakest surgeons. A mannequin such as HEAD START provides a more ethical alternative than live patients for surgeons who need more practice.

Our study results were less conclusive regarding the utility of extended HEAD START training at measurably improving surgeon skill during live surgeries, as the study was not powered to measure change in surgeon skill. At the end of the study, on average, surgeons who practiced on the simulator had only slightly more improved skills when performing live surgery compared with surgeons who did not have additional simulation practice (mean change 1.3 in EHS vs. 0.5 in SHS). Numerous studies have attempted to understand whether surgeon improvement using a simulator corresponds with improvement in the operating room, with mixed results [13–15]. Many factors influence whether skills on a simulator translate to live surgery skills, and appropriate validation of evaluation tools is one major component [16,17]. A validated assessment tool helps confirm that the trainee and trainer have the same perspective of the simulated surgery and establishes a quantifiable outcome [17]. Future research should focus on refining and validating the assessments used to evaluate trichiasis surgeon competence.

Challenges identified by the study team underscored the logistical difficulty of implementing such a comprehensive intervention. Because surgeons typically operate in remote areas while the trainers were based in the capital, cartridges had to be transferred to trainers in Addis Ababa. This is a barrier in regions where mail service is unreliable or unavailable, and it adds an extra cost for the program. In our study, the cartridge delivery process was often slow and resulted in delays before surgeons could receive feedback on their simulated surgeries. Multiple surgeons felt that this additional cost for transportation needed to be addressed before scaling up the program. Our study was not designed to evaluate the cost-effectiveness of incorporating long-term simulation practice into trachoma control programs. Further, multiple approaches could be used to integrate simulation practice, making it difficult to assign accurate costs to this approach on a large-scale basis. The largest cost of the simulator is the reusable base; additional eyelid cartridges cost approximately $15 each. Future studies should examine the marginal costs of adding extended HEAD START under different scenarios, including shared use of simulation bases or centralized monthly practice.

Further, this study required significant investment from an ophthalmologist trainer, evidenced by one ophthalmologist who was unable to fully fulfill their duties during this pilot study. Recruiting ophthalmologists to review cartridges, coordinate calls with surgeons, and conduct feedback sessions with surgeons may not be feasible when scaling-up this extended training. However, despite this sizable commitment, trainers still thought the opportunity for continued interaction with surgeons was beneficial. The WHO also recommends continued monitoring and audit of TT surgeons [18]. As countries make steps towards trachoma elimination, surgeons may operate less frequently with longer breaks between periods of higher surgical productivity. Additional HEAD START practice has already been demonstrated to have significant benefit on postoperative outcomes when used as a refresher training for experienced surgeons: in one study, refresher training incorporating HEAD START significantly reduced one-year PTT rates from nearly 29% to 16% [19]. While that study did not assess regular simulator usage, it suggests that further surgical simulation and feedback from trainers positively impact longer-term outcomes.

Interestingly, surgeon improvement was consistent regardless of whether surgeons received monthly feedback from an ophthalmologist trainer, indicating that extended practice on the HEAD START device might provide a benefit on its own. This finding is consistent with a 2011 review that found inconclusive evidence to support supervision for medical interventions in low- and middle-income countries [20]. When establishing audits and extended surgeon trainings, it will be important to weigh the positive surgeon perception of feedback with the limited improvement in surgical skill associated with ophthalmologist feedback. Further research is needed to tease out the most impactful implementation of supportive supervision for TT surgeons.

Interpretation of our study findings is limited by small sample size and lack of power to conduct hypothesis testing regarding changes in surgeon skill. While a lack of statistical testing leaves readers unsure how to interpret the reported data, p values are unlikely to be representative in a small population [21]. Small pilot studies are better used to assess feasibility than to generate effect measures [22]. We were also unable to thoroughly assess the impact of the frequency of performing live surgeries during the study period on final surgical skill. We found limited differences in follow-up assessment outcomes based on surgical volume for both groups. However, it is important to note that nearly half of the surgeons performed fewer than 20 surgeries during the study period. Thus, it is difficult to determine whether performing more surgeries impacted follow-up surgical skill assessment. Certainly, other surgical fields have shown that high-volume surgeons often have better outcomes than low-volume surgeons [23]. However, such studies typically did not assess starting surgeon skill. It is possible that individuals with significant innate skill are more likely to progress to high-volume surgeons.

Finally, as the survey was not administered by a third party, surgeons may have felt pressured to report favorable opinions about the HEAD START training. During initial trainings, surgeons were instructed that we sought their honest feedback about program feasibility; surgeons ultimately reported both positive and negative opinions about the program, suggesting they felt comfortable responding truthfully.

## Conclusions

Extended training with the HEAD START surgical simulator may provide new TT surgeons with an opportunity to gain confidence and skill. Both surgeon trainees and trainers had overwhelmingly positive feedback about the opportunity for additional practice. When incorporated with trichiasis surgery training, extended HEAD START practice has the potential to hone surgeons’ skills beyond the traditional training model. Future studies are needed to establish the feasibility of trainer feedback components, to validate assessment tools, and to evaluate whether additional training impacts live surgical outcomes.

## Supporting information

S1 File LTHS_Form A. Script for monthly phone call with trainees.
(DOCX)

S2 File LTHS_Form B. Surgical Skills Assessment.
(DOCX)

S3 File LTHS_Form C. End of project questionnaire for trainees.
(DOC)

S4 File LTHS_Form D. End of project questionnaire for trainers.
(DOCX)
