# Supplementary material for: Trachomatous trichiasis surgeons appreciate using HEAD START for extended training during periods of low surgical activity: A preliminary study
Source: PLoS Negl Trop Dis. 2026 Feb 26;20(2):e0013948. doi: 10.1371/journal.pntd.0013948 (PMC12978490; doi:10.1371/journal.pntd.0013948)
Supplement: S1 File — Script for monthly phone call with trainees. (DOCX) [file pntd.0013948.s001.docx]

**Monthly Trainer Call**

1. How many cartridges has the trainee/surgeon sent for review since the previous call?

______________________________________________________

1. Rank the trainee/surgeon on each skill below. Select the average score across all cartridges:

1: very poor 2: poor 3: good 4: very good 5: excellent

Very Poor Excellent

Ability to make a straight incision 1 2 3 4 5

Ability to take proper bites 1 2 3 4 5

Ability to evenly space sutures 1 2 3 4 5

Ability to tie knots well 1 2 3 4 5

**General Assessment:**

1. What does the trainee/surgeon need to focus on for the next month?

______________________________________________________

______________________________________________________

______________________________________________________

1. In what areas is the trainee/surgeon performing well?

________________________________________________________________________

______________________________________________________

______________________________________________________

1. Any feedback or general thoughts for the trainee/surgeon:

______________________________________________________

______________________________________________________

______________________________________________________

1. Agreed-upon steps for the trainee/surgeon before next month’s call:

______________________________________________________

______________________________________________________

______________________________________________________
