# Supplementary material for: Trachomatous trichiasis surgeons appreciate using HEAD START for extended training during periods of low surgical activity: A preliminary study
Source: PLoS Negl Trop Dis. 2026 Feb 26;20(2):e0013948. doi: 10.1371/journal.pntd.0013948 (PMC12978490; doi:10.1371/journal.pntd.0013948)
Supplement: S2 File — Surgical Skills Assessment. (DOCX) [file pntd.0013948.s002.docx]

**Surgical Skills Assessment**

1. Please check one:

□ Initial assessment

□ Post-rainy season assessment

1. Rank the trainee/surgeon on each skill below:

1: very poor 2: poor 3: good 4: very good 5: excellent

Very Poor Excellent

Ability to inject anesthesia 1 2 3 4 5

Ability to place the eversion suture properly 1 2 3 4 5

Ability to place the Trabut plate properly (including eversion) 1 2 3 4 5

Ability to hold and manipulate instruments 1 2 3 4 5

Ability to make a straight incision 1 2 3 4 5

Ability to take proper bites 1 2 3 4 5

Ability to evenly space sutures 1 2 3 4 5

Ability to tie knots well 1 2 3 4 5

Ability to follow the appropriate logical order and technique 1 2 3 4 5

Ability to maintain sterility 1 2 3 4 5

1. Compared with all individuals you have previously trained, how would you rate the trainee’s overall innate skill level for performing trichiasis surgery?

The best I have seen

Top 10%

Top 25%

About average

Below average

Very low skill level

Aspects of innate skill to consider:

- Ability to hold and manipulate instruments
- Ability to make a straight incision
- Ability to evenly space sutures
- The way knots are tied for suturing
- Ability to follow the appropriate logical order and technique

1. How many eyes did you assess during this evaluation?_______
2. Comments:
