# Supplementary material for: Trachomatous trichiasis surgeons appreciate using HEAD START for extended training during periods of low surgical activity: A preliminary study
Source: PLoS Negl Trop Dis. 2026 Feb 26;20(2):e0013948. doi: 10.1371/journal.pntd.0013948 (PMC12978490; doi:10.1371/journal.pntd.0013948)
Supplement: S3 File — End of project questionnaire for trainees. (DOC) [file pntd.0013948.s003.doc]

**End of Project Questionnaire for Trainees**

1. What did you like about practicing regularly on HEAD START?
2. What did you dislike about practicing regularly on HEAD START?
3. Did you find the monthly conference calls useful? Explain your answer.
4. For the future, what things should we keep as part of regular HEAD START practice?
5. What things should we change about the process?
6. Is 2 cartridges weekly a sufficient number of cartridges to maintain skill levels during periods of inactivity (such as the rainy season)? If not, how many cartridges should be practiced weekly during periods of surgical inactivity?
7. During periods when you are regularly performing surgery, do you feel it is useful to practice with HEAD START? Explain.
8. Please provide any additional comments on why you did or did not like regular use of HEAD START and/or monthly calls with a senior surgeon.
